# High Procalcitonin Does Not Always Indicate a Bacterial Infection

**DOI:** 10.7759/cureus.72274

**Published:** 2024-10-24

**Authors:** Hamza Shahzad, Adan J Khokhar, Saad Ibrahim, Mian U Farooq, Rimsha Rashid, Hameed U Raheem, Gurjit Singh

**Affiliations:** 1 Medicine, Doncaster Royal Infirmary, Doncaster, GBR; 2 General and Specialist Medicine, The Royal Oldham Hospital, Oldham, GBR; 3 Medicine, Wythenshawe Hospital, Wythenshawe, GBR; 4 Medicine, University Hospital Crosshouse, Kilmarnock, GBR

**Keywords:** bacterial infection, inflammatory markers, metastatic disease, procalcitonin, thyroid medullary cancer

## Abstract

Procalcitonin (PCT) has become essential for differentiating bacterial infections from viral infections and noninfectious causes of inflammation, as most inflammatory markers rise with inflammation without indicating a specific etiology. The significance of PCT was underscored during the COVID-19 pandemic, when many patients exhibited elevated inflammatory markers, complicating decisions regarding antibacterial therapy without PCT levels. However, a rise in PCT cannot always be attributed to a bacterial infection, as it is also a precursor of calcitonin produced in the thyroid gland. We present a case of a 77-year-old female patient with a history of medullary thyroid cancer, which she underwent surgical resection and radiotherapy for in 1980. She also experienced right vocal cord palsy as a side effect of radiotherapy and had stable liver metastases. Her past medical history included hypothyroidism, trigeminal neuralgia, gastroesophageal reflux disease, prediabetes, meningioma, vertebral fracture, osteoporosis, depression, and chronic kidney disease stage 4. The patient had recurrent episodes of aspiration pneumonia and poor swallowing. She presented with progressive dysphagia, and her chest X-ray revealed consolidation, with positive Mycoplasma IgM. At the end of her antibiotic course, there were no residual infective symptoms. Prior to admission, a CT scan of the thorax, abdomen, and pelvis showed bilateral upper zone medial fibrotic changes related to radiation, with no sinister lung lesions. It also revealed a few non-united fractures involving the left-sided ribs posteriorly, while biliary distension and liver and bone disease appeared stable. Interestingly, her PCT levels remained consistently elevated at >100 ng/L throughout her admission, despite normal CRP and white blood cell counts. This case was extensively discussed with the infectious diseases team, who suggested that the elevated PCT levels were likely related to thyroid cancer metastases, which can synthesize PCT. Consequently, PCT would be functionally increased in such circumstances and would be an unreliable marker for infection. Further analysis indicated that the PCT elevation resulted from her stable medullary thyroid cancer liver metastases, which were dormant and not affecting liver function but were secreting PCT. This case illustrates that a patient with medullary thyroid cancer metastases to the liver, who was treated for pneumonia, exhibited persistently high PCT levels despite completing the treatment. Calcitonin levels, checked on one occasion, were also elevated, reinforcing that the rise in PCT was attributed to production from medullary cancer metastatic cells rather than an inflammatory response. In bacterial septicemia, PCT is produced through alternate pathways, either directly or indirectly, and is therefore not related to the rise in calcitonin. Consequently, persistently high PCT levels in the absence of other infection markers should prompt further investigation.

## Introduction

Procalcitonin (PCT) has emerged as a widely recognized biomarker for diagnosing and guiding antibiotic therapy in bacterial infections. Typically, PCT levels increase significantly during bacterial infections, making it an essential tool for differentiating bacterial causes from viral or noninfectious sources of inflammation. Additionally, PCT serves as a precursor to calcitonin, a hormone produced by the thyroid gland that plays a crucial role in calcium homeostasis.

In the context of a bacterial infection, PCT is produced in increased amounts outside the thyroid gland, particularly in response to cytokine and endotoxin release, as demonstrated in this study [1]. This highlights the intricate relationship between the thyroid and PCT. Therefore, PCT functions not only as a marker of systemic inflammation but also as a component of the normal physiological processes within the thyroid gland, as noted in this study [2].

## Case presentation

The patient had a background of medullary thyroid cancer, diagnosed in 1980, for which she underwent surgical resection and radiotherapy. She had stable liver metastasis, bone issues, and right vocal cord palsy as a side effect of the radiotherapy. Her medical history included hypothyroidism, trigeminal neuralgia, gastroesophageal reflux disease, prediabetes, vaginal dryness, meningioma, vertebral fracture, osteoporosis, depression, and chronic kidney disease stage 4. She experienced recurrent aspiration pneumonia and had difficulties swallowing, necessitating Botox injections to the mandibular gland to reduce excessive salivation.

On April 4, 2024, a 77-year-old female patient was admitted with progressive dysphagia over five days, leading to difficulty swallowing saliva and shortness of breath. Excess salivation was noted despite a recent Botox injection in March 2024. An upper gastrointestinal endoscopy and ENT review did not reveal any new findings.

Chest X-ray showed progressive consolidation, and she tested positive for Mycoplasma IgM on April 4, 2024. At this stage, urinary *Legionella* and pneumococcal antigens, COVID-19 tests, and blood cultures were negative. She initially required oxygen to meet target saturation on April 5, 2024, but was successfully weaned off by April 12, 2024. Treatment for pneumonia was completed with a 10-day course of doxycycline. There were no residual infective symptoms at the end of the antibiotic course, as her initial shortness of breath and oxygen requirements resolved.

Prior to her current admission, a CT of the thorax, abdomen, and pelvis did not show any sinister lung parenchymal changes. There were bilateral upper zone medial fibrotic changes consistent with previous radiation, with no pleural nodularity or areas of thickening. A few non-united fractures were noted involving the left-sided ribs posteriorly. The liver disease appeared stable, and the bone disease was also stable, with biliary distension remaining unchanged.

Interestingly, her PCT levels had been greater than 100 ng/mL throughout her admission episode (Table 1).

**Table 1:** Serial inflammatory investigations during admission CRP reference range: 0-5 mg/L; WCC reference range: 4-12 × 10^9^/L. PCT, procalcitonin; WCC, white cell count

Sample collected date	PCT value (ng/mL)	CRP (mg/L)	WCC (× 10^9^/L)
April 4, 2024	>100	<1.0	5.1
April 5, 2024	>100	<1.0	7
April 12, 2024	>100	7.92	5.9
April 27, 2024	>100	11.82	8.7
May 9, 2024	-	2.45	7.9

## Discussion

The above was discussed with an infectious diseases consultant, who suggested that a persistent rise in PCT could probably be related to thyroid cancer. The medullary cells in the thyroid gland synthesize PCT; thus, this can be functionally increased in these circumstances, as noted in this study [3]. Therefore, this would be an unreliable marker of infection in this case.

**Figure 1:**
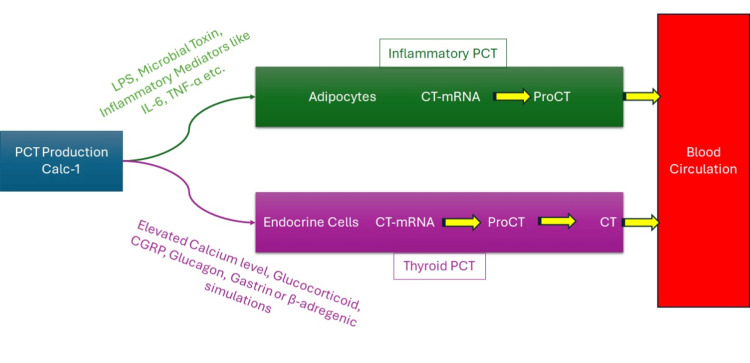
Two different pathways of PCT synthesis in the body PCT, procalcitonin Image credit: Saad Ibrahim

On further analysis, PCT levels were elevated because the patient had medullary thyroid cancer with liver metastasis. Despite thyroid resection, the metastases in the liver were dormant and not affecting the liver itself; however, they were secreting PCT, as indicated in this study [4]. PCT is produced in thyroid C cells, from a CALC-1 gene located on chromosome 11. The mRNA product is known as pre-PCT, which is further modified to a 116-amino acid PCT. Finally, it is cleaved into three distinct molecules: active calcitonin (32 amino acids), keta-calcitonin (21 amino acids), and N-terminal PCT (57 amino acids). In bacterial septicemia, PCT is produced via alternate pathways (Figure 1), either directly or indirectly; hence, it is not related to the rise in calcitonin, as discussed in this study [5].

## Conclusions

This case involved a patient with medullary thyroid cancer metastasizing to the liver, who was treated for pneumonia, yet her PCT levels remained elevated even after completing the treatment. The elevated PCT was linked to production by the metastatic cells of medullary cancer rather than any inflammatory response, as there are two distinct and entirely segregated pathways for PCT secretion in the body. Therefore, a persistent rise in PCT, particularly in the absence of other inflammatory markers or overt signs of bacterial infection, should prompt further investigations.
